# Spatial Multiplexing of Whispering Gallery Mode Sensors

**DOI:** 10.3390/s23135925

**Published:** 2023-06-26

**Authors:** Stephen Holler, Matthew Speck

**Affiliations:** Department of Physics and Engineering Physics, Fordham University, Bronx, NY 10458, USA

**Keywords:** whispering gallery mode, resonator, photonic sensor, morphology-dependent resonance, microcavity, HPV, virus

## Abstract

Whispering gallery mode resonators have proven to be robust and sensitive platforms for the trace detection of chemical and/or biological analytes. Conventional approaches using serially addressed resonators face challenges in simultaneous multi-channel (i.e., multi-species) detection. We present an alternative monitoring scheme that allows for the spatial multiplexing of whispering gallery mode resonators with the simultaneous observation of the resonance spectra from each of them. By imaging arrays of microspheres and monitoring the glare spot intensities through image processing routines, resonance spectra from multiple resonators may be simultaneously recorded without interference or confounding effects of serial excitation/detection. We demonstrate our multiplexed imaging approach with bulk refractive index variations and virus–antibody binding.

## 1. Introduction

The rise of and the subsequent pandemic associated with SARS-CoV-2, the causative agent for COVID-19 [1], has raised public awareness of the plethora of variants that may exist for a single biological agent, let alone the myriad pathogens that exist in the world. The development of compact, robust sensors for the detection and identification of pathogens serves both diagnostic and defense purposes. Furthermore, the ability to test for multiple agents is vital for the successful deployment of a sensor technology. Optical sensors offer the requisite properties [2], and the development of integrated photonic platforms allows for compact sensors that may be mass-produced and readily deployed [3].

Photonic sensors based on resonant optical cavities are particularly attractive. However, those integrated photonics circuits that utilize optical waveguides may suffer from detection challenges when multiple resonators are serially addressed. The density of resonators on a single channel is constrained by the ability to unmix the signals. For the same detection capabilities, sparse resonator arrangements may be offset by increased photonic channels. Although feasible, this arrangement adds to the complexity of the platform.

One way to circumvent the limitations of serial coupling is to switch coupling schemes and implement an imaging stage using low-cost CCD detectors for parallel, spatially multiplexed detection [4]. In what follows, we will discuss this approach and demonstrate its ability to obtain resonance spectra from optical microcavities. Furthermore, we will demonstrate the sensing modality by altering the refractive index of the bulk medium, as well as showing spectral changes due to virus adsorption on the microcavity surface. We will first review the underlying optical phenomenon and describe the experimental apparatus.

## 2. The Whispering Gallery Mode Biosensor

Whispering gallery modes (WGMs), also known as morphology-dependent resonances (MDRs) [5], have a long and varied history in mediating processes in optical microcavities, from aerosol chemistry [6,7,8,9] to lasers [10,11,12] to innovative sensor platforms [13,14,15,16,17]. These resonances emerge from Mie’s solution to the problem of the light scattering of a plane wave by a sphere [18]. However, generalizations of the problem to both non-plane-wave illumination and non-spherical geometries also produce morphology-dependent resonances. For this paper, we will only consider the spherically symmetric geometry. The solution to the boundary value problem of electromagnetic waves interacting with and within a spherical microcavity satisfies the Helmholtz equation:

(1) $$\nabla^{2}\vec{E}+k^{2}\vec{E}=0,$$

where $\vec{E}$ is the electric field, *k* (=2π/λ) is the wavenumber of the light, and λ is the wavelength. Specifically, the resonances correspond to the poles of the expansion coefficients and may be in either the transverse electric (TE) or transverse magnetic (TM) polarization state. However, this can be physically unsatisfying, and it is useful to visualize the problem in a slightly different manner. Using vector spherical harmonics to obtain the solution, one finds that the radial component of the electric field ($\psi_{r}$) satisfies

(2) $$\frac{d^{2}\psi_{r}}{dr^{2}}=\left\{k_{0}^{2}-\left[k_{0}^{2}\left(1-n{\left(r\right)}^{2}\right)+\frac{\ell \left(\ell +1\right)}{r^{2}}\right]\right\}\psi_{r}=0,$$

where $k_{0}$ is the free space wavenumber, n(r) is the refractive index of the sphere of radius *a*, and *ℓ* is the angular mode number [19].

It should be noted that Equation (2) resembles the Schrödinger equation for the hydrogen atom. Because of this analogy, the spherical microcavity has come to be known as a photonic atom, with the resonant modes being equivalent to the bound states of the atom, and this model provides a useful picture in considering the resonant modes in a spherical optical resonator. These modes are characterized by their polarization (TE or TM) and three quantum numbers: the angular mode number (*ℓ*), the radial mode number (ν), and the azimuthal mode number (*m*). Photons “bound” in these resonant modes are trapped within the effective potential well described by

(3) $$V\left(r\right)=k_{0}^{2}\left(1-n{\left(r\right)}^{2}\right)+\frac{\ell \left(\ell +1\right)}{r^{2}}.$$

**Figure 1:**
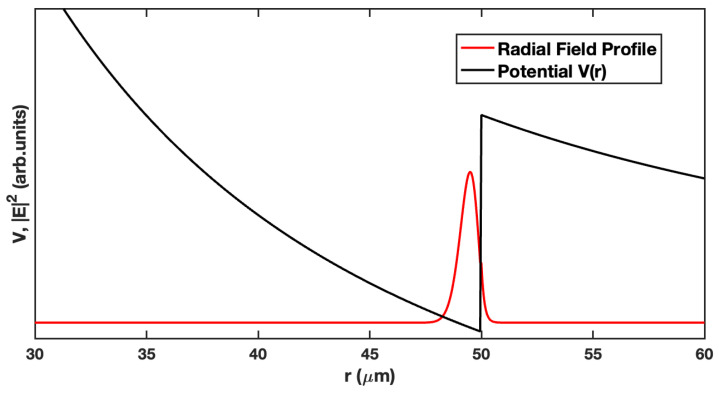
An illustration of a first-radial-order WGM bound state (red curve) within the potential well (black curve) given by Equation (3) with the parameters *$k_{0}$a* = 394.439, *ℓ* = 575, *n* = 1.46. The evanescent tail of the mode can be seen decaying away beyond the surface of the microsphere. This tail reaches out into the surrounding medium, permitting it to interact with nearby analytes, resulting in a shift in the resonance wavelength.

This is illustrated in Figure 1, which shows the potential well (Equation (3), black curve) and a first-order radial mode (red curve) for an air-clad microsphere (*a* = 50 μm) with a size parameter *$k_{0}$a* = 394.439, a refractive index *n* = 1.46, and an angular mode number *ℓ* = 575.

This photonic atom analogy is appealing because it allows one to think of the whispering gallery modes in a familiar quantum mechanical sense. The radial mode is confined within the potential well and extends beyond the barrier (surface of the microsphere, *r* = 50 μm in Figure 1) by a short distance. This evanescent field is able to interact with the external environment, allowing the resonant mode to be perturbed by local environmental changes. These changes may be effected by bulk refractive index variations or surface interactions such as hybridization or antibody–antigen binding. These interactions alter the resonance condition, resulting in a shift in the resonant wavelength.

The evanescent field decays exponentially from the resonator surface, so that, as previously mentioned, the electric field may be perturbed by interactions with the local environment near the surface of the microsphere. Consider the case of a nearby/bound target analyte. When a dielectric particle enters the evanescent field of the WGM, it becomes polarized by the electric field, and the microcavity must give up some energy in order for this to happen. By considering the fractional change in the energy of the system, an expression for the shift in the resonance frequency/wavelength due to the interaction can be obtained [13]:

(4) $$\frac{\Delta E}{E}=\frac{\Delta \omega }{\omega }=-\frac{\Delta \lambda }{\lambda }=-\frac{ℜ\left[\alpha_{ex}\right]{\left|E_{0}\left(r_{a}\right)\right|}^{2}}{2\int \epsilon \left(r_{c}\right){\left|E_{0}\left(r_{c}\right)\right|}^{2}dV_{c}}.$$

Here, the numerator represents the energy required to polarize the analyte, where $ℜ[\alpha_{ex}]$ is the real part of the excess polarizability (i.e., the difference between the polarizability of the target and the surrounding medium), and $E_{0}\left(r_{a}\right)$ is the electric field at the position of the analyte. The denominator represents the total energy stored within the cavity. For low-order radial modes, more than 95% of the WGM energy is contained within the microcavity, with only a small fraction extending beyond the surface [20].

Equation (4) is known as the Reactive Sensing Principle (RSP) [13] and is applicable to other morphologies and materials, such as disks and toroids [21,22,23,24,25,26]. Since it was first introduced, the RSP has proven robust, exhibiting excellent correspondence with a wide range of experimental results [13,27,28,29,30,31].

## 3. Experimental Apparatus

The evanescent field extends through the potential barrier in Figure 1, allowing the mode to be accessed externally. There are several different coupling schemes to stimulate the resonant modes. The most common approaches use either bus waveguide coupling [32] or prism coupling [33].

Waveguide coupling enables the development of photonic circuits and the serial multiplexing of resonators but requires a cladding-free waveguide. For lithographically patterned waveguides, this is feasible, but, in addition to being cost-prohibitive for many labs, the control of the waveguide–resonator gap, and hence the coupling constant, can present challenges. Tapered fibers have a low barrier to entry, providing a relatively inexpensive means of coupling light from an optical fiber to resonators of different geometries. However, tapered fibers require good control of the tapering process to produce fibers having the correct diameter for proper phase matching [34], and they are easily broken through handling.

Prism coupling relies on the overlap of the evanescent field from a beam undergoing total internal reflection (TIR) with the extended tail of the resonant mode. Light from the TIR is frustrated, allowing photons to tunnel through the barrier (Figure 1) and populate the cavity mode. We opted to use this approach for its simplicity, robustness, and ability to stimulate WGMs in a parallel rather than serial fashion, such as that afforded by the bus waveguide.

**Figure 2:**
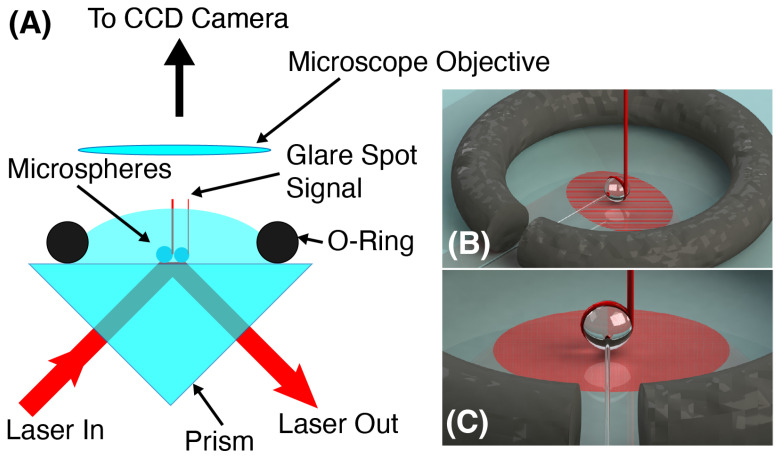
(**A**) Microspherical resonators are positioned atop a prism at the location where the tunable laser strikes the surface from the underside. The light undergoes total internal reflection and the tail of the evanescent field allows light to couple with the whispering gallery modes of the microspheres. An o-ring around the spheres defines the sample volume, which is filled with solution. A microscope looks from above to image the microsphere array and records light emission at the glare spots on the edge of the resonators on a CCD camera (not shown). (**B**,**C**) show two different renderings of a single microsphere inside the o-ring sample volume and held in position by the fiber stem. The red band around the sphere is the light circulating in the resonance, while the beam directed upward is the glare spot signal.

Figure 2A shows a cartoon of the experimental setup that we employed. Light from a tunable distributed feedback (DFB) laser operating at 780 nm is fired into a right-angle prism perpendicular to one face. The light undergoes TIR at the top surface and exits the prism through the other face. At the spot where the laser reflects, an evanescent field is produced, extending beyond the top surface of the prism. Our approach positions one or several microspheres on the prism surface, directly above the location where the TIR occurs. The field from the laser is then able to couple to the WGMs of the microspherical resonators. On resonance, the coupling is strong and the light entering the mode circumnavigates the microspheres close to the surface. As the light circulates, it leaks out tangentially from the edge of the microsphere. Looking from above with a microscope and CCD camera, one can image the microspheres and monitor the leaking light. On resonance, the glare spots on the edge of the microsphere will intensify due to the resonant buildup of energy within the microcavity. By monitoring where in the tuning cycle the glare spots intensify, we can determine changes in the resonance condition due to environmental interactions. Some prism coupling schemes observe the coupling via the transmitted signal, which manifests as dips in the resonant wavelengths in the same way as for bus waveguide coupling [35]. We opted for a different approach, imaging the microspheres from above and observing the leakage signal from light coupled to the resonances. This approach is similar to one that has been employed in fluorescence-based WGM measurements [36,37].

Since we are ultimately targeting biological analytes, we must form a vessel to contain the sample solution. The sample is held fixed over the illuminating spot with an o-ring that sits atop the prism surface [38]. A gap is cut into the o-ring to permit the microspheres, held in place by the fiber stem, to be positioned above the TIR spot, while surface tension prevents the solution from leaking out of the o-ring container. Figure 2B,C shows renderings of a single microsphere within the sample volume bound by an o-ring. The fiber stem, which allows for the easy positioning of the microsphere, is clearly visible in these renderings. The red band around the sphere and the red tube tangential to the edge represent light circulating in the WGM and the glare spot signal, respectively.

The microspheres used in these experiments were fabricated from a segment of single-mode optical fiber (SMF-28e). The polymer jacket was removed and the end of the fiber was rotated in the beam of a 10 W CO${}_{2}$ laser (10.6 μm). The strong absorption by the fiber melts the glass to form a ball at the end of the fiber stem. This produces a high-quality microspherical cavity. For our experiments, the fiber stem was held in a fiber mount affixed to the 3-axis translation stage, which allowed the microspheres to be accurately positioned within the sample volume.

**Figure 3:**
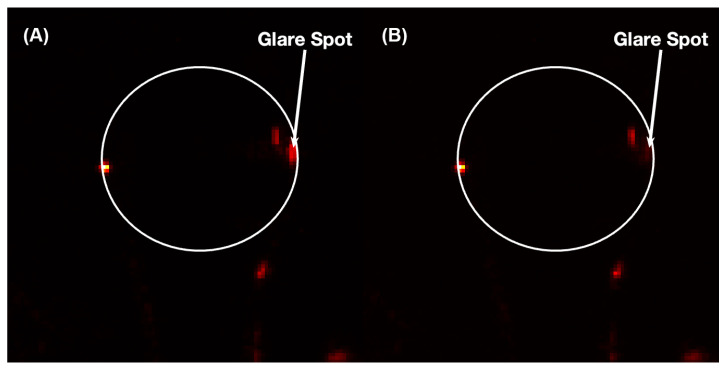
Images of a prism-coupled microsphere: (**A**) on-resonance and (**B**) off-resonance. The glare spot indicated results from leakage of the circulating light. This is the region monitored to produce the resonance spectrum.

A microscope was positioned above the prism surface, where one or several microspheres were located within the sensing region. The tunable DFB laser light couples to the microsphere, resulting in photons being injected into the resonant modes. As this light circumnavigates the microsphere, it slowly leaks out tangential to the surface [39,40]. The leakage at one edge of the microsphere is directed upward towards the microscope (Figure 2), which is collected and imaged onto a CCD camera. The image of the microsphere shows a bright “glare spot” on the edge that varies in intensity as the laser is tuned through WGM resonances. Figure 3 shows images of a prism-coupled microsphere at two different times in the tuning cycle. Figure 3A is on-resonance and shows a bright glare spot due to the leakage from the circulating resonant light. Figure 3B corresponds to the off-resonance condition and one can see that the glare spot intensity is significantly diminished. Such glare spots may be used to perform spectroscopy within droplets [41], but, in this case, the spectral signature of the microsphere is determined by monitoring the intensity of these spots. A wide field of view allows multiple microspheres to be located within the beam spot on the prism surface and monitored simultaneously, i.e., spatial multiplexing.

The DFB laser was tuned using a virtual instrument constructed in LabView 2012. This code was used to synchronously tune the laser and capture an image. The resulting image was stored to disk for subsequent analysis within MatLab R2022. The image sequences were loaded into MatLab and, within each image, the pixels associated with the bright spot on the edge of the microsphere were identified. The glare spot signal was integrated over a pixel region (typically 3 × 3) to produce a resonance spectrum for the microsphere being monitored.

## 4. Results and Discussion

Experiments were performed to demonstrate the feasibility of recording resonance spectra from single, and simultaneously from multiple, microsphere arrangements by imaging the glare spots. In addition, we examined the shifts in the spectra due to changes to the local environment following changes in the bulk refractive index and viral adsorption.

The tunable 780 nm DFB laser was fired into the prism as depicted in Figure 2A, producing a several mm${}^{2}$ elliptical spot on the surface that served as the coupling region. The spheres were affixed by their stems to separate translation stages and positioned over the coupling region. Individually, they were lowered until scattered laser light was observed on the camera, indicating that they were interacting with the evanescent field. They were positioned within an o-ring that acted as the sample container [38]. Prior to performing any data acquisition, the laser was tuned and the spheres were observed to “blink” as the laser was scanned through WGM resonances.

**Figure 4:**
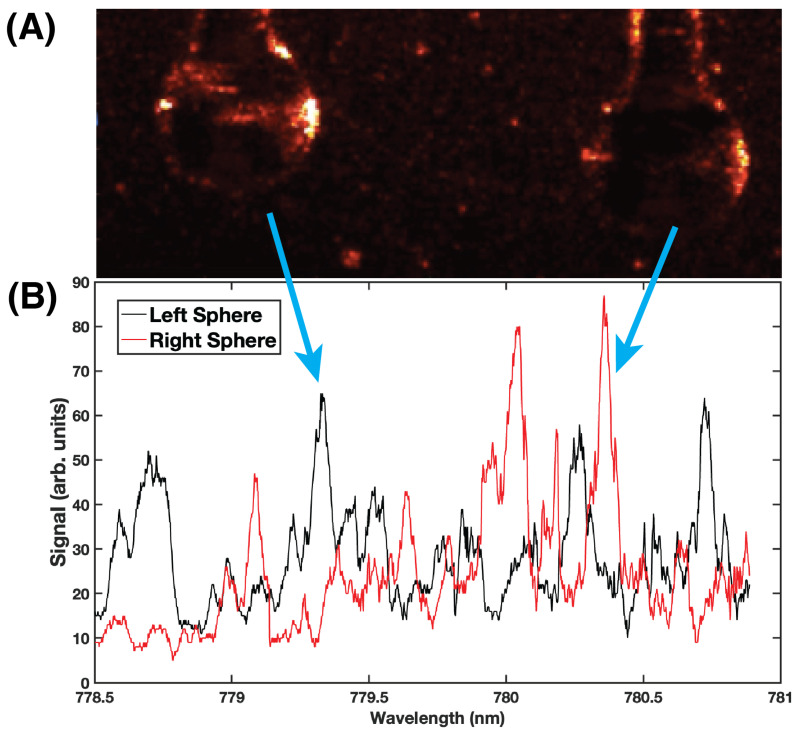
(**A**) Image showing two microspheres situated on the prism and illuminated by the evanescent field of the tunable DFB laser. The sphere on the left shows a bright spot on the right edge corresponding to light leakage from a WGM resonance. At this point, the sphere on the right is not in resonance and does not exhibit a bright spot on its right edge. (**B**) WGM resonance spectra obtained simultaneously by imaging two spatially separated microspheres. Both spectra exhibit narrow peaks that may be used for trace species detection in the WGM biosensor. A video of two spheres undergoing resonance as the laser is tuned may be downloaded from the Supplementary Materials.

Figure 4A shows a recorded image of two microspheres situated on the prism and illuminated by the DFB laser. At this time in the scan cycle, the sphere on the left shows a bright spot on the right edge of the sphere. This corresponds to the leakage towards the CCD camera of the light circulating within the WGM resonance. Note that the sphere on the right is not in resonance and therefore does not exhibit a bright spot on its edge. There is some background scattering observable from the surface of the prism and the microsphere stems.

The spectra obtained from the two spheres in Figure 4A are shown in Figure 4B. The spectrum from the left sphere (black curve) shows several prominent peaks within the tuning range. The spectrum from the right sphere (red curve) also has a number of prominent peaks, along with a couple of peaks that appear narrow. Some overlap of resonance modes from the two different microspheres is observed. The spatial multiplexing imaging approach has the advantage of being able to discriminate the different modes, unlike the serial configuration. If the microspheres were inline on a bus waveguide, then their spectra would confound each other, making discrimination and sensing difficult. Even if resonances were associated with specific microspheres *a priori*, once analytes interact with the evanescent field and the resonances shift (Equation (4)), the WGMs could/would move through each other, prohibiting accurate measurements.

**Figure 5:**
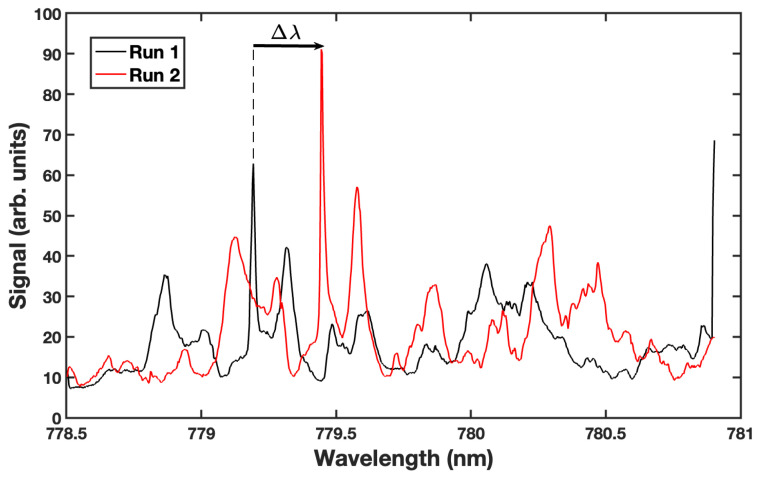
Resonance spectra from a single 250 μm diameter sphere immersed in water (Run 1, black) and then a water/methanol solution (Run 2, red). The change in the bulk refractive index surrounding the sphere results in a ∼0.26 nm shift in the WGM resonances. For clarity, an arrow indicates the shift in one of the resonances. Videos of the two runs, in which the sphere is observed to resonate as the laser is tuned, may be downloaded from the Supplementary Materials.

Figure 5 shows the resonance spectra from two different experiments on the same 250 μm diameter microsphere. In the first run, the microsphere was immersed in water and the resonance spectrum was obtained by monitoring the glare spot intensity (black curve). A 50 μL solution of water/methanol (50/50 by volume) was injected into the sample volume. This solution was allowed to diffuse and thermally equilibrate with the water initially in the sample volume. A short time later, a second spectrum was taken (red curve), showing that the resonances had undergone a shift. Sharp resonance peaks were observed during both runs, the narrowest having a Q value of $4\times 10^{4}$. The change in the surrounding refractive index affected the resonance condition of the microsphere, resulting in a ∼0.26 nm shift in the spectrum. This shift was determined by computing the shifts in several resonances. For clarity, an arrow indicating Δλ for one resonance is shown in Figure 5.

**Figure 6:**
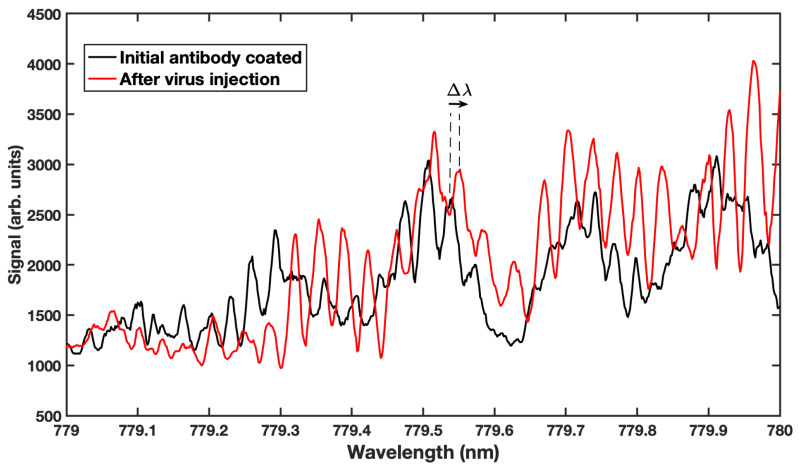
Resonance spectra showing the effect of viral binding to an antibody-coated microsphere. The black curve in the WGM spectrum was taken prior to the introduction of a Human Papillomavirus solution. After five minutes of diffusion through the sample cell and binding to the microsphere surface, the red curve was recorded. A definite shift is observed due to the response of the microsphere to the mass loading of the virus.

In addition to monitoring changes in the bulk solution, the whispering gallery mode biosensor has been used to detect the adsorption of target biomolecules. Figure 6 shows the WGM resonance spectrum (black curve) of a microsphere coated with antibodies that bind to Human Papillomavirus (HPV). Shortly after this spectrum was recorded, a 1 μL solution of HPV, which the antibodies were designed to bind with, was injected into the sample volume and allowed to diffuse to the microsphere. The specific binding events altered the resonance condition of the system, resulting in a shift in the resonance peaks. In essence, the virus “grows” a layer over the surface of the sphere, effectively increasing the size of the sphere so that, in order to remain on resonance, the mode wavelength shifts to longer values. The red curve shows the WGM resonance spectrum approximately 5 min post-injection. At this point in time, the resonances have shifted 0.12 nm due to the binding of the virus to the antibodies on the surface, in accordance with the RSP (Equation (4)). For clarity, an arrow indicating Δλ for one resonance is shown in Figure 6. The shift in this spectrum is smaller than that observed in Figure 5, owing to the thin layer of viral material aggregating on the surface of the microsphere.

## 5. Summary

We have demonstrated the feasibility of performing spectral measurements of WGM resonances by imaging spatially multiplexed microspherical cavities. In addition to performing single sphere measurements, this work showed that changes in the local environment, whether bulk refractive index changes or the adsorption of biological particles, can be readily observed with this approach, thus offering another tool for the optical detection of an array of pathogens.

## Data Availability

Data are available upon request.

## Supplementary Materials

The following supporting information can be downloaded at: https://www.mdpi.com/article/10.3390/s23135925/s1, Videos for the data shown in Figure 4 can be found as 2sphere.mp4. The videos for the two runs shown in Figure 5 may be downloaded as Run1b.mp4 for the first run and Run2b.mp4 for the second run.

## Abbreviations

The following abbreviations are used in this manuscript:

DFB	Distributed Feedback
HPV	Human Paplillomavirus
MDR	Morphology-Dependent Resonance
RSP	Reactive Sensing Principle
TIR	Total Internal Reflection
WGM	Whispering Gallery Mode

## References

[1] Fauci A.S., Lane H.C., Redfield R.R. (2020). COVID-19—Navigating the Uncharted. N. Engl. J. Med..

[2] Ferreira M.F.S., Castro-Camus E., Ottaway D.J., López-Higuera J.M., Feng X., Jin W., Jeong Y., Picqué N., Tong L., Reinhard B.M. (2017). Roadmap on optical sensors. J. Opt..

[3] Liao Z., Zhang Y., Li Y., Miao Y., Gao S., Lin F., Deng Y., Geng L. (2019). Microfluidic chip coupled with optical biosensors for simultaneous detection of multiple analytes: A review. Biosens. Bioelectron..

[4] Holler S., Speck M., Vo-Dinh T., Lieberman R.A., Gauglitz G.G. (2016). Spatial multiplexing of whispering gallery mode sensors for trace species detection. Proceedings of the Advanced Environmental, Chemical, and Biological Sensing Technologies XIII.

[5] Hill S.C., Benner R.E., Barber P.W., Chang R.K. (1988). Morphology-Dependent Resonances. Optical Effects Associated with Small Particles.

[6] Zhang J.X., Aiello D., Aker P.M. (1994). A spectroscopic tour through the liquid aerosol interface: Implications for atmospheric chemistry. J. Geophys. Res. Atmos..

[7] Arnold S., Holler S., Druger S.D. (1996). Imaging enhanced energy transfer in a levitated aerosol particle. J. Chem. Phys..

[8] Mitchem L., Buajarern J., Ward A.D., Reid J.P. (2006). A Strategy for Characterizing the Mixing State of Immiscible Aerosol Components and the Formation of Multiphase Aerosol Particles through Coagulation. J. Phys. Chem. B.

[9] Moridnejad A., Preston T.C., Krieger U.K. (2017). Tracking Water Sorption in Glassy Aerosol Particles using Morphology-Dependent Resonances. J. Phys. Chem. A.

[10] Qian S.X., Snow J.B., Tzeng H.M., Chang R.K. (1986). Lasing Droplets: Highlighting the Liquid-Air Interface by Laser Emission. Science.

[11] Taniguchi H., Tomisawa H., Kido J. (1995). Ultra-low-threshold europium chelate laser in morphology-dependent resonances. Appl. Phys. Lett..

[12] Wei G.Q., Wang X.D., Liao L.S. (2020). Recent Advances in Organic Whispering-Gallery Mode Lasers. Laser Photonics Rev..

[13] Arnold S., Khoshsima M., Teraoka I., Holler S., Vollmer F. (2003). Shift of whispering-gallery modes in microspheres by protein adsorption. Opt. Lett..

[14] Ioppolo T., Ayaz U.K., Ötügen M.V. (2009). High-resolution force sensor based on morphology dependent optical resonances of polymeric spheres. J. Appl. Phys..

[15] Rahman A. (2011). Temperature sensor based on dielectric optical microresonator. Opt. Fiber Technol..

[16] Ali A.R., Ioppolo T. (2014). Effect of Angular Velocity on Sensors Based on Morphology Dependent Resonances. Sensors.

[17] Xia R.L., Liu B., Hu Y., Liu J., Fu Y., He X.D., Lu P., Farrell G., Yuan J., Wu Q. (2023). Rapid Detection of SARS-CoV-2 Nucleocapsid Protein by a Label-free biosensor based on Optical Fiber Cylindrical Micro-resonator. IEEE Sens. J..

[18] Mie G. (1908). Beiträge zur Optik trüber Medien, speziell kolloidaler Metallösungen. Ann. Phys..

[19] Johnson B.R. (1993). Theory of morphology-dependent resonances: Shape resonances and width formulas. J. Opt. Soc. Am. A.

[20] Chowdhury D.Q., Hill S.C., Mazumder M.M. (1993). Quality factors and effective-average modal gain or loss in inhomogeneous spherical resonators: Application to two-photon absorption. IEEE J. Quantum Electron..

[21] Zhu J., Özdemir S.K., He L., Chen D.R., Yang L. (2011). Single virus and nanoparticle size spectrometry by whispering-gallery-mode microcavities. Opt. Exp..

[22] Stoian R.I., Bui K., Rosenberger A. (2015). Silica hollow bottle resonators for use as whispering gallery mode based chemical sensors. J. Opt..

[23] Malmir K., Habibiyan H., Ghafoorifard H. (2016). An ultrasensitive optical label-free polymeric biosensor based on concentric triple microring resonators with a central microdisk resonator. Opt. Commun..

[24] Ghali H., Chibli H., Nadeau J.L., Peter Y.A. (2016). Real-Time Detection of Staphylococcus Aureus Using Whispering Gallery Mode Optical Microdisks. Biosensors.

[25] Ajad A.K., Islam J., Kaysir R., Atai J. (2021). Highly sensitive bio sensor based on WGM ring resonator for hemoglobin detection in blood samples. Optik.

[26] Suebka S., Nguyen P.D., Gin A., Su J. (2021). How Fast It Can Stick: Visualizing Flow Delivery to Microtoroid Biosensors. ACS Sens..

[27] Vollmer F., Arnold S. (2008). Whispering-gallery-mode biosensing: Label-free detection down to single molecules. Nat. Meth..

[28] Dantham V.R., Holler S., Kolchenko V., Wan Z., Arnold S. (2012). Taking whispering gallery-mode single virus detection and sizing to the limit. Appl. Phys. Lett..

[29] Keng D., Tan X., Arnold S. (2014). Whispering gallery micro-global positioning system for nanoparticle sizing in real time. Appl. Phys. Lett..

[30] Foreman M.R., Keng D., Treasurer E., Lopez J.R., Arnold S. (2017). Whispering gallery mode single nanoparticle detection and sizing: The validity of the dipole approximation. Opt. Lett..

[31] Graybill R.M., Para C.S., Bailey R.C. (2016). PCR-free, Multiplexed Expression Profiling of microRNAs using Silicon Photonic Microring Resonators. Anal. Chem..

[32] Serpengüzel A., Griffel G., Arnold S. (1995). Excitation of resonances of microspheres on an optical fiber. Opt. Lett..

[33] Gorodetsky M., Ilchenko V. (1994). High-Q optical whispering-gallery microresonators: Precession approach for spherical mode analysis and emission patterns with prism couplers. Opt. Comm..

[34] Knight J.C., Cheung G., Jacques F., Birks T.A. (1997). Phase-matched excitation of whispering-gallery-mode resonances by a fiber taper. Opt. Lett..

[35] Pan Y.L., Chang R.K. (2003). Highly efficient prism coupling to whispering gallery modes of a square *μ*-cavity. Appl. Phys. Lett..

[36] Huckabay H.A., Dunn R.C. (2011). Whispering gallery mode imaging for the multiplexed detection of biomarkers. Sens. Actuators B Chem..

[37] Huckabay H.A., Wildgen S.M., Dunn R.C. (2013). Label-free detection of ovarian cancer biomarkers using whispering gallery mode imaging. Biosens. Bioelectron..

[38] Vollmer F., Arnold S., Keng D. (2008). Single virus detection from the reactive shift of a whispering-gallery mode. Proc. Natl. Acad. Sci. USA.

[39] Garrett C.G.B., Kaiser W., Bond W.L. (1961). Stimulated. Emission into Optical Whispering Modes of Spheres. Phys. Rev..

[40] Ashkin A., Dziedzic J.M. (1981). Observation of optical resonances of dielectric spheres by light scattering. Appl. Opt..

[41] Arnold S., Hill S.C., Holler S., Li J.H., Serpengüzel A., Auffermann W.F. (1995). Aerosol particle microphotography and glare-spot absorption spectroscopy. Opt. Lett..

